# Ethyl 2-(4-carb­oxy­benzyl­idene)-7-methyl-3-oxo-5-phenyl-2,3-dihydro-5*H*-thia­zolo[3,2-*a*]pyrimidine-6-carboxyl­ate–*N*,*N*-dimethyl­formamide (1/1)

**DOI:** 10.1107/S1600536812000050

**Published:** 2012-01-18

**Authors:** Noor Afshan Banu, V. Bheema Raju

**Affiliations:** a Department of Chemistry, KNS Institute of Technology, Bangalore 560 064, India; b Department of Chemistry, Dr. Ambedkar Institute of Technology, Bangalore 560 056, India

## Abstract

In the title compound, C_24_H_20_N_2_O_5_S·C_3_H_7_NO, a benzene ring is positioned axially to the pyrimidine ring, which adopts a twist-boat conformation, and is inclined to its mean plane by 85.36 (7)°. In the crystal, inter­molecular C—H⋯O inter­actions result in centrosymmetric head-to-head dimers with an *R*_2_^2^(14) graph-set motif along the *b* axis. Pairs of C—H⋯O and O—H⋯O hydrogen bonds form centrosymmetric head-to-head dimers about inversion centres, corresponding to an *R*_2_^2^(7) graph-set motif along the *a* axis.

## Related literature

For pharmacological properties of pyrimidine derivatives and general background, see: Alam *et al.* (2010 ▶). For a related structure, see: Jotani *et al.* (2010 ▶). For graph-set motifs, see: Bernstein *et al.* (1995 ▶). For puckering parameters, see: Cremer & Pople (1975 ▶).
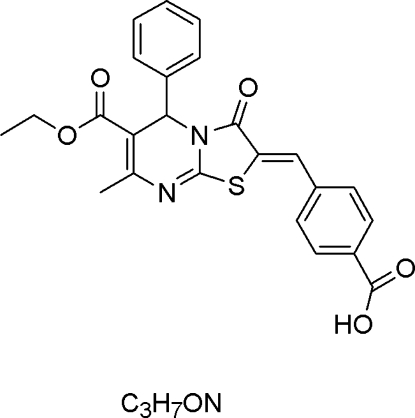


## Experimental

### 

#### Crystal data

C_24_H_20_N_2_O_5_S·C_3_H_7_NO
*M* *_r_* = 521.58
Triclinic, 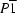

*a* = 8.4146 (4) Å
*b* = 12.0699 (5) Å
*c* = 14.0185 (6) Å
α = 71.799 (2)°
β = 78.753 (2)°
γ = 86.488 (2)°
*V* = 1326.56 (10) Å^3^
*Z* = 2
Mo *K*α radiation
μ = 0.17 mm^−1^
*T* = 296 K
0.20 × 0.20 × 0.15 mm

#### Data collection

Bruker SMART APEX CCD detector diffractometer
Absorption correction: multi-scan (*SADABS*; Bruker, 1998 ▶) *T*_min_ = 0.967, *T*_max_ = 0.975
23685 measured reflections
5788 independent reflections
3560 reflections with *I* > 2σ(*I*)’
*R*_int_ = 0.033

#### Refinement

*R*[*F* ^2^ > 2σ(*F* ^2^)] = 0.058
*wR*(*F* ^2^) = 0.178
*S* = 1.04
5788 reflections
339 parameters
H-atom parameters constrained
Δρ_max_ = 0.39 e Å^−3^
Δρ_min_ = −0.29 e Å^−3^

### 

Data collection: *SMART* (Bruker, 1998 ▶); cell refinement: *SAINT-Plus* (Bruker, 1998 ▶); data reduction: *SAINT-Plus*; program(s) used to solve structure: *SHELXS97* (Sheldrick, 2008 ▶); program(s) used to refine structure: *SHELXL97* (Sheldrick, 2008 ▶); molecular graphics: *ORTEP-3* (Farrugia, 1997 ▶) and *CAMERON* (Watkin *et al.*, 1996 ▶); software used to prepare material for publication: *WinGX* (Farrugia, 1999 ▶).

## Supplementary Material

Crystal structure: contains datablock(s) global, I. DOI: 10.1107/S1600536812000050/pv2495sup1.cif

Structure factors: contains datablock(s) I. DOI: 10.1107/S1600536812000050/pv2495Isup2.hkl

Supplementary material file. DOI: 10.1107/S1600536812000050/pv2495Isup3.cml

Additional supplementary materials:  crystallographic information; 3D view; checkCIF report

## Figures and Tables

**Table 1:** Hydrogen-bond geometry (Å, °)

*D*—H⋯*A*	*D*—H	H⋯*A*	*D*⋯*A*	*D*—H⋯*A*
C81—H81⋯O5^i^	0.93	2.32	3.053 (3)	135
O4—H4⋯O11^ii^	0.82	1.81	2.618 (3)	170
C17—H17⋯O3^iii^	0.93	2.43	3.310 (3)	157

Symmetry codes: (i) 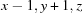; (ii) 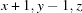; (iii) 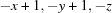.

**Fig. 1.**
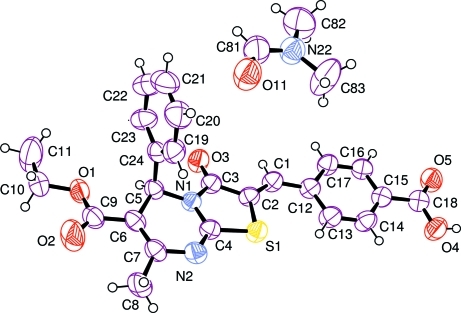
ORTEP (Farrugia, 1997) view of the title compound, showing 50% probability ellipsoids and the atom numbering scheme.

**Fig. 2.**
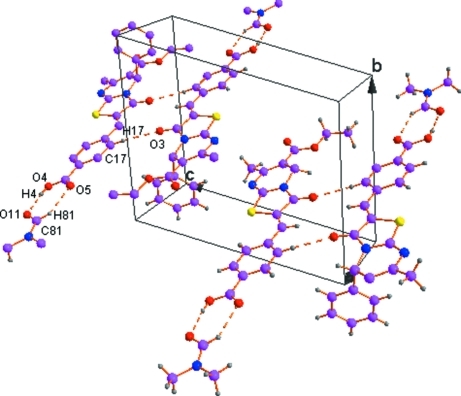
A unit cell packing of the title compound showing intermolecular interactions with dotted lines.

## supplementary crystallographic information

### Comment

Pyrimidine derivatives are of interest because of their pharmacological properties (Alam *et al.*, 2010). In the title molecule (Fig. 1), the central pyrimidine ring with a chiral C5 atom is significantly puckered and adopts a conformation which is best described as an intermediate between a boat and a screw boat form as reported in a closely related structure (Jotani *et al.*, 2010). The ring puckering parameters (Cremer & Pople, 1975) for the pyrimidine ring are *Q*(2) = 0.1985 (2) Å, φ(2) = 159.70 (7)° and θ= 70.74 (6)°. A mean planes calculation shows that the atoms C5 and N1 deviate from the mean plane of the remaining ring atoms (N2/C4/C6/C7) by -0.1442 (2) and -0.0949 (2) Å, respectively, indicating that the conformation of the ring is that of a twisted boat. In the molecule, the fused thiazolopyrimidine and the benzene ring (C19—C24) are almost orthogonal with the dihedral angle between these rings being 85.36 (7)°. In the crystal, the carbonyl O3 atom is involved in a hydrogen bonding interaction C17—H17···O3 forming centrosymmetric dimers with *R*^2^_2_(14) graph-set motif (Bernstein *et al.*, 1995) along the *b*-axis. Pairs of C81—H81···O5 and O4—H4···O11 intermolecular interections generate centrosymmetric head-to-head dimers about inversion centres, corresponding to an *R*^2^_2_(7) graph-set motif along the *a*- axis.

### Experimental

A mixture of 5-phenyl-6-methyl-2-thioxo-1,2,3,4-tetrahydro -pyrimidine-5-carboxylic acid ethyl ester (0.01 mol), chloroacetic acid (0.01 mol), 4-carboxy benzaldehyde (0.01 mol) and sodium acetate (1.5 g) in a mixture of glacial acetic acid and acetic anhydride (25 ml, 1:1) was refluxed for 8–10 h. The reaction mixture was concentrated and the solid thus obtained was filtered and recrystallized from ethyl acetate to get the title compound (78% yield, m.p. 427–428 K). The compound was recrystallized by slow evaporation of ethyl acetate-ethanol (6:4) solution, yielding pale yellow single crystals suitable for X-ray diffraction.

### Refinement

The H atoms were placed at calculated positions in the riding model approximation with O—H = 0.82 Å and C—H = 0.93, 0.96, 0.97 and 0.98 Å, for aryl, methyl, methylene and methyne type H-atoms, respectively, with *U*_iso_(H) = 1.5*U*_eq_(C-methyl) or 1.2*U*_eq_(O/C non-methyl).

### Crystal data

**Table:**

C_24_H_20_N_2_O_5_S·C_3_H_7_NO	*Z* = 2
*M**_r_* = 521.58	*F*(000) = 548
Triclinic, *P*1	*D*_x_ = 1.306 Mg m^−^^3^
Hall symbol: -P 1	Mo *K*α radiation, λ = 0.71073 Å
*a* = 8.4146 (4) Å	Cell parameters from 5788 reflections
*b* = 12.0699 (5) Å	θ = 1.6–27.0°
*c* = 14.0185 (6) Å	µ = 0.17 mm^−^^1^
α = 71.799 (2)°	*T* = 296 K
β = 78.753 (2)°	Block, yellow
γ = 86.488 (2)°	0.20 × 0.20 × 0.15 mm
*V* = 1326.56 (10) Å^3^	

### Data collection

**Table:**

Bruker SMART APEX CCD detector diffractometer	5788 independent reflections
Radiation source: fine-focus sealed tube	3560 reflections with *I* > 2σ(*I*)'
graphite	*R*_int_ = 0.033
ω scans	θ_max_ = 27.0°, θ_min_ = 1.6°
Absorption correction: multi-scan (*SADABS*; Bruker, 1998)	*h* = −10→8
*T*_min_ = 0.967, *T*_max_ = 0.975	*k* = −15→14
23685 measured reflections	*l* = −17→17

### Refinement

**Table:**

Refinement on *F*^2^	Primary atom site location: structure-invariant direct methods
Least-squares matrix: full	Secondary atom site location: difference Fourier map
*R*[*F*^2^ > 2σ(*F*^2^)] = 0.058	Hydrogen site location: inferred from neighbouring sites
*wR*(*F*^2^) = 0.178	H-atom parameters constrained
*S* = 1.04	*w* = 1/[σ^2^(*F*_o_^2^) + (0.103*P*)^2^] where *P* = (*F*_o_^2^ + 2*F*_c_^2^)/3
5788 reflections	(Δ/σ)_max_ < 0.001
339 parameters	Δρ_max_ = 0.39 e Å^−^^3^
0 restraints	Δρ_min_ = −0.29 e Å^−^^3^

### Special details

**Table:**

Geometry. All s.u.'s (except the s.u. in the dihedral angle between two l.s. planes) are estimated using the full covariance matrix. The cell s.u.'s are taken into account individually in the estimation of s.u.'s in distances, angles and torsion angles; correlations between s.u.'s in cell parameters are only used when they are defined by crystal symmetry. An approximate (isotropic) treatment of cell s.u.'s is used for estimating s.u.'s involving l.s. planes.
Refinement. Refinement of *F*^2^ against ALL reflections. The weighted *R*-factor *wR* and goodness of fit *S* are based on *F*^2^, conventional *R*-factors *R* are based on *F*, with *F* set to zero for negative *F*^2^. The threshold expression of *F*^2^ > 2σ(*F*^2^) is used only for calculating *R*-factors(gt) *etc*. and is not relevant to the choice of reflections for refinement. *R*-factors based on *F*^2^ are statistically about twice as large as those based on *F*, and *R*- factors based on ALL data will be even larger.

### Fractional atomic coordinates and isotropic or equivalent isotropic displacement parameters (Å^2^)

**Table:**

	*x*	*y*	*z*	*U*_iso_*/*U*_eq_	
N22	0.6187 (3)	0.93566 (19)	0.32137 (16)	0.0806 (6)	
C81	0.4744 (4)	0.9872 (3)	0.3273 (2)	0.0860 (8)	
H81	0.4495	1.0414	0.2685	0.103*	
C82	0.7324 (4)	0.9640 (3)	0.2250 (2)	0.1124 (11)	
H82A	0.6825	1.0170	0.1725	0.169*	
H82B	0.7631	0.8939	0.2074	0.169*	
H82C	0.8270	0.9997	0.2315	0.169*	
C83	0.6722 (5)	0.8554 (3)	0.4099 (3)	0.1303 (14)	
H83A	0.6222	0.8755	0.4699	0.195*	
H83B	0.7878	0.8600	0.4014	0.195*	
H83C	0.6420	0.7774	0.4170	0.195*	
O11	0.3700 (2)	0.96682 (18)	0.40786 (15)	0.1004 (6)	
S1	0.30622 (7)	0.34995 (5)	0.35943 (4)	0.0618 (2)	
N1	0.1227 (2)	0.48407 (14)	0.24302 (12)	0.0505 (4)	
N2	0.0013 (2)	0.40960 (16)	0.41686 (12)	0.0610 (5)	
O1	−0.29300 (18)	0.64562 (14)	0.17146 (12)	0.0718 (5)	
O2	−0.4299 (2)	0.5986 (2)	0.33005 (16)	0.1173 (8)	
O3	0.27644 (19)	0.52114 (14)	0.08332 (11)	0.0702 (5)	
O5	1.2299 (2)	0.16084 (16)	0.22813 (13)	0.0837 (5)	
O4	1.0939 (2)	0.07417 (16)	0.38392 (13)	0.0839 (5)	
H4	1.1835	0.0469	0.3928	0.126*	
C1	0.5294 (3)	0.38515 (19)	0.17748 (16)	0.0588 (6)	
H1	0.5440	0.4212	0.1073	0.071*	
C2	0.3822 (3)	0.40097 (18)	0.22858 (15)	0.0538 (5)	
C3	0.2609 (3)	0.47380 (18)	0.17423 (15)	0.0536 (5)	
C4	0.1213 (3)	0.42052 (18)	0.34338 (15)	0.0527 (5)	
C5	−0.0051 (2)	0.56909 (17)	0.21469 (15)	0.0514 (5)	
H5	−0.0318	0.5646	0.1510	0.062*	
C6	−0.1543 (3)	0.53676 (19)	0.29785 (16)	0.0562 (5)	
C7	−0.1450 (3)	0.46191 (19)	0.39185 (15)	0.0579 (6)	
C8	−0.2833 (3)	0.4227 (2)	0.47932 (17)	0.0775 (7)	
H8A	−0.2833	0.4658	0.5264	0.116*	
H8B	−0.2722	0.3410	0.5135	0.116*	
H8C	−0.3834	0.4361	0.4543	0.116*	
C9	−0.3069 (3)	0.5942 (2)	0.27206 (18)	0.0668 (6)	
C10	−0.4341 (3)	0.7095 (2)	0.1380 (2)	0.0848 (8)	
H10A	−0.4629	0.7697	0.1708	0.102*	
H10B	−0.5257	0.6572	0.1556	0.102*	
C11	−0.3925 (5)	0.7620 (4)	0.0270 (3)	0.1493 (17)	
H11A	−0.2973	0.8093	0.0103	0.224*	
H11B	−0.4809	0.8097	0.0030	0.224*	
H11C	−0.3721	0.7015	−0.0049	0.224*	
C12	0.6698 (3)	0.32199 (18)	0.21211 (15)	0.0565 (5)	
C13	0.6715 (3)	0.2478 (2)	0.31156 (17)	0.0687 (6)	
H13	0.5766	0.2368	0.3602	0.082*	
C14	0.8092 (3)	0.1918 (2)	0.33831 (17)	0.0662 (6)	
H14	0.8070	0.1426	0.4045	0.079*	
C15	0.9529 (3)	0.20750 (18)	0.26740 (16)	0.0575 (5)	
C16	0.9541 (3)	0.2815 (2)	0.16903 (16)	0.0626 (6)	
H16	1.0498	0.2931	0.1210	0.075*	
C17	0.8164 (3)	0.3372 (2)	0.14224 (16)	0.0631 (6)	
H17	0.8198	0.3865	0.0760	0.076*	
C18	1.1052 (3)	0.1454 (2)	0.29103 (18)	0.0642 (6)	
C19	0.0875 (3)	0.7233 (2)	0.2777 (2)	0.0732 (7)	
H19	0.0673	0.6714	0.3436	0.088*	
C20	0.1494 (4)	0.8341 (2)	0.2594 (3)	0.0925 (9)	
H20	0.1719	0.8557	0.3136	0.111*	
C21	0.1769 (3)	0.9101 (3)	0.1647 (3)	0.0941 (9)	
H21	0.2184	0.9836	0.1536	0.113*	
C22	0.1446 (4)	0.8801 (2)	0.0860 (3)	0.0948 (9)	
H22	0.1636	0.9335	0.0207	0.114*	
C23	0.0835 (3)	0.7708 (2)	0.10042 (18)	0.0736 (7)	
H123	0.0609	0.7513	0.0453	0.088*	
C24	0.0565 (2)	0.69169 (18)	0.19627 (16)	0.0563 (5)	

### Atomic displacement parameters (Å^2^)

**Table:**

	*U*^11^	*U*^22^	*U*^33^	*U*^12^	*U*^13^	*U*^23^
N22	0.0713 (15)	0.0885 (15)	0.0760 (14)	0.0075 (11)	−0.0098 (11)	−0.0205 (12)
C81	0.0765 (19)	0.099 (2)	0.0758 (18)	0.0023 (16)	−0.0131 (15)	−0.0185 (15)
C82	0.096 (2)	0.136 (3)	0.093 (2)	−0.003 (2)	0.0108 (18)	−0.035 (2)
C83	0.138 (3)	0.138 (3)	0.092 (2)	0.052 (2)	−0.030 (2)	−0.007 (2)
O11	0.0815 (14)	0.1229 (17)	0.0802 (13)	0.0172 (11)	−0.0075 (11)	−0.0152 (11)
S1	0.0619 (4)	0.0721 (4)	0.0442 (3)	0.0072 (3)	−0.0036 (2)	−0.0126 (3)
N1	0.0504 (10)	0.0541 (10)	0.0437 (9)	0.0008 (8)	−0.0037 (7)	−0.0136 (7)
N2	0.0576 (12)	0.0769 (13)	0.0442 (10)	−0.0033 (9)	0.0006 (8)	−0.0182 (9)
O1	0.0460 (9)	0.0916 (12)	0.0690 (10)	0.0024 (8)	−0.0089 (7)	−0.0137 (8)
O2	0.0598 (12)	0.172 (2)	0.0863 (13)	0.0252 (12)	0.0123 (10)	−0.0116 (13)
O3	0.0627 (10)	0.0922 (12)	0.0420 (8)	0.0120 (8)	0.0000 (7)	−0.0091 (8)
O5	0.0639 (11)	0.1041 (14)	0.0731 (11)	0.0089 (9)	−0.0061 (9)	−0.0185 (9)
O4	0.0758 (12)	0.0851 (12)	0.0725 (11)	0.0157 (10)	−0.0111 (9)	−0.0025 (9)
C1	0.0627 (14)	0.0618 (13)	0.0480 (11)	0.0066 (10)	−0.0040 (10)	−0.0163 (10)
C2	0.0579 (13)	0.0552 (12)	0.0446 (11)	0.0009 (10)	−0.0025 (9)	−0.0144 (9)
C3	0.0528 (12)	0.0596 (13)	0.0449 (11)	0.0011 (9)	−0.0006 (9)	−0.0167 (10)
C4	0.0566 (13)	0.0578 (13)	0.0428 (11)	−0.0036 (10)	−0.0052 (9)	−0.0162 (9)
C5	0.0454 (11)	0.0616 (13)	0.0460 (10)	−0.0023 (9)	−0.0043 (9)	−0.0170 (9)
C6	0.0476 (12)	0.0684 (14)	0.0536 (12)	−0.0072 (10)	−0.0014 (9)	−0.0234 (11)
C7	0.0493 (13)	0.0750 (15)	0.0512 (12)	−0.0068 (10)	−0.0005 (9)	−0.0260 (11)
C8	0.0581 (15)	0.111 (2)	0.0544 (13)	−0.0087 (13)	0.0049 (11)	−0.0200 (13)
C9	0.0487 (13)	0.0808 (16)	0.0644 (14)	−0.0059 (11)	−0.0017 (11)	−0.0171 (12)
C10	0.0514 (15)	0.0922 (19)	0.099 (2)	0.0032 (13)	−0.0153 (13)	−0.0124 (16)
C11	0.096 (3)	0.213 (4)	0.095 (2)	0.045 (3)	−0.021 (2)	0.009 (3)
C12	0.0574 (13)	0.0597 (13)	0.0496 (11)	0.0056 (10)	−0.0006 (10)	−0.0197 (10)
C13	0.0628 (15)	0.0707 (15)	0.0581 (13)	0.0114 (11)	0.0076 (11)	−0.0127 (11)
C14	0.0710 (16)	0.0635 (14)	0.0541 (13)	0.0105 (11)	−0.0035 (11)	−0.0111 (11)
C15	0.0604 (14)	0.0545 (13)	0.0604 (13)	0.0045 (10)	−0.0100 (10)	−0.0232 (10)
C16	0.0619 (14)	0.0734 (15)	0.0491 (12)	0.0004 (11)	0.0002 (10)	−0.0205 (11)
C17	0.0643 (15)	0.0748 (15)	0.0456 (11)	0.0054 (11)	−0.0046 (10)	−0.0161 (10)
C18	0.0657 (16)	0.0651 (15)	0.0643 (14)	0.0028 (11)	−0.0126 (12)	−0.0238 (12)
C19	0.0745 (17)	0.0701 (16)	0.0810 (17)	−0.0003 (12)	−0.0282 (13)	−0.0235 (13)
C20	0.094 (2)	0.0787 (19)	0.121 (3)	−0.0065 (16)	−0.0330 (19)	−0.0448 (19)
C21	0.079 (2)	0.0646 (18)	0.132 (3)	−0.0110 (14)	0.0014 (18)	−0.0317 (19)
C22	0.101 (2)	0.0659 (18)	0.091 (2)	−0.0015 (15)	0.0207 (17)	−0.0094 (15)
C23	0.0810 (17)	0.0666 (16)	0.0605 (14)	−0.0029 (13)	0.0094 (12)	−0.0148 (12)
C24	0.0385 (11)	0.0596 (13)	0.0665 (13)	0.0034 (9)	−0.0004 (9)	−0.0197 (11)

### Geometric parameters (Å, °)

**Table:**

N22—C81	1.330 (3)	C6—C9	1.475 (3)
N22—C83	1.447 (3)	C7—C8	1.493 (3)
N22—C82	1.450 (3)	C8—H8A	0.9600
C81—O11	1.254 (3)	C8—H8B	0.9600
C81—H81	0.9300	C8—H8C	0.9600
C82—H82A	0.9600	C10—C11	1.463 (4)
C82—H82B	0.9600	C10—H10A	0.9700
C82—H82C	0.9600	C10—H10B	0.9700
C83—H83A	0.9600	C11—H11A	0.9600
C83—H83B	0.9600	C11—H11B	0.9600
C83—H83C	0.9600	C11—H11C	0.9600
S1—C2	1.743 (2)	C12—C17	1.399 (3)
S1—C4	1.747 (2)	C12—C13	1.404 (3)
N1—C4	1.374 (2)	C13—C14	1.363 (3)
N1—C3	1.384 (2)	C13—H13	0.9300
N1—C5	1.464 (3)	C14—C15	1.388 (3)
N2—C4	1.274 (3)	C14—H14	0.9300
N2—C7	1.404 (3)	C15—C16	1.388 (3)
O1—C9	1.336 (3)	C15—C18	1.486 (3)
O1—C10	1.451 (3)	C16—C17	1.362 (3)
O2—C9	1.194 (3)	C16—H16	0.9300
O3—C3	1.207 (2)	C17—H17	0.9300
O5—C18	1.214 (3)	C19—C24	1.384 (3)
O4—C18	1.306 (3)	C19—C20	1.395 (3)
O4—H4	0.8200	C19—H19	0.9300
C1—C2	1.339 (3)	C20—C21	1.343 (4)
C1—C12	1.447 (3)	C20—H20	0.9300
C1—H1	0.9300	C21—C22	1.342 (4)
C2—C3	1.473 (3)	C21—H21	0.9300
C5—C6	1.514 (3)	C22—C23	1.387 (4)
C5—C24	1.526 (3)	C22—H22	0.9300
C5—H5	0.9800	C23—C24	1.369 (3)
C6—C7	1.359 (3)	C23—H123	0.9300
			
C81—N22—C83	122.3 (3)	O2—C9—O1	121.6 (2)
C81—N22—C82	120.1 (2)	O2—C9—C6	127.1 (2)
C83—N22—C82	117.5 (3)	O1—C9—C6	111.30 (19)
O11—C81—N22	123.6 (3)	O1—C10—C11	107.4 (2)
O11—C81—H81	118.2	O1—C10—H10A	110.2
N22—C81—H81	118.2	C11—C10—H10A	110.2
N22—C82—H82A	109.5	O1—C10—H10B	110.2
N22—C82—H82B	109.5	C11—C10—H10B	110.2
H82A—C82—H82B	109.5	H10A—C10—H10B	108.5
N22—C82—H82C	109.5	C10—C11—H11A	109.5
H82A—C82—H82C	109.5	C10—C11—H11B	109.5
H82B—C82—H82C	109.5	H11A—C11—H11B	109.5
N22—C83—H83A	109.5	C10—C11—H11C	109.5
N22—C83—H83B	109.5	H11A—C11—H11C	109.5
H83A—C83—H83B	109.5	H11B—C11—H11C	109.5
N22—C83—H83C	109.5	C17—C12—C13	117.0 (2)
H83A—C83—H83C	109.5	C17—C12—C1	117.96 (19)
H83B—C83—H83C	109.5	C13—C12—C1	125.00 (19)
C2—S1—C4	91.62 (10)	C14—C13—C12	121.4 (2)
C4—N1—C3	115.96 (18)	C14—C13—H13	119.3
C4—N1—C5	120.81 (16)	C12—C13—H13	119.3
C3—N1—C5	122.69 (16)	C13—C14—C15	120.5 (2)
C4—N2—C7	116.87 (17)	C13—C14—H14	119.8
C9—O1—C10	115.94 (19)	C15—C14—H14	119.8
C18—O4—H4	109.5	C16—C15—C14	119.0 (2)
C2—C1—C12	131.4 (2)	C16—C15—C18	118.1 (2)
C2—C1—H1	114.3	C14—C15—C18	122.9 (2)
C12—C1—H1	114.3	C17—C16—C15	120.5 (2)
C1—C2—C3	120.46 (19)	C17—C16—H16	119.7
C1—C2—S1	128.94 (18)	C15—C16—H16	119.7
C3—C2—S1	110.56 (14)	C16—C17—C12	121.5 (2)
O3—C3—N1	123.1 (2)	C16—C17—H17	119.2
O3—C3—C2	126.56 (19)	C12—C17—H17	119.2
N1—C3—C2	110.27 (17)	O5—C18—O4	123.2 (2)
N2—C4—N1	125.9 (2)	O5—C18—C15	121.9 (2)
N2—C4—S1	122.68 (16)	O4—C18—C15	114.9 (2)
N1—C4—S1	111.41 (15)	C24—C19—C20	119.0 (3)
N1—C5—C6	108.64 (16)	C24—C19—H19	120.5
N1—C5—C24	109.51 (16)	C20—C19—H19	120.5
C6—C5—C24	113.05 (16)	C21—C20—C19	120.8 (3)
N1—C5—H5	108.5	C21—C20—H20	119.6
C6—C5—H5	108.5	C19—C20—H20	119.6
C24—C5—H5	108.5	C22—C21—C20	120.2 (3)
C7—C6—C9	122.49 (19)	C22—C21—H21	119.9
C7—C6—C5	121.1 (2)	C20—C21—H21	119.9
C9—C6—C5	116.33 (19)	C21—C22—C23	121.0 (3)
C6—C7—N2	122.32 (18)	C21—C22—H22	119.5
C6—C7—C8	126.0 (2)	C23—C22—H22	119.5
N2—C7—C8	111.64 (19)	C24—C23—C22	119.6 (3)
C7—C8—H8A	109.5	C24—C23—H123	120.2
C7—C8—H8B	109.5	C22—C23—H123	120.2
H8A—C8—H8B	109.5	C23—C24—C19	119.3 (2)
C7—C8—H8C	109.5	C23—C24—C5	121.2 (2)
H8A—C8—H8C	109.5	C19—C24—C5	119.48 (19)
H8B—C8—H8C	109.5		
			
C83—N22—C81—O11	−3.7 (5)	C10—O1—C9—O2	1.9 (4)
C82—N22—C81—O11	179.8 (3)	C10—O1—C9—C6	−177.28 (19)
C12—C1—C2—C3	177.9 (2)	C7—C6—C9—O2	12.6 (4)
C12—C1—C2—S1	0.5 (4)	C5—C6—C9—O2	−165.1 (3)
C4—S1—C2—C1	178.1 (2)	C7—C6—C9—O1	−168.28 (19)
C4—S1—C2—C3	0.47 (15)	C5—C6—C9—O1	14.0 (3)
C4—N1—C3—O3	177.18 (19)	C9—O1—C10—C11	178.0 (3)
C5—N1—C3—O3	−11.1 (3)	C2—C1—C12—C17	−169.5 (2)
C4—N1—C3—C2	−4.2 (2)	C2—C1—C12—C13	9.1 (4)
C5—N1—C3—C2	167.44 (16)	C17—C12—C13—C14	−1.0 (4)
C1—C2—C3—O3	2.6 (3)	C1—C12—C13—C14	−179.7 (2)
S1—C2—C3—O3	−179.55 (19)	C12—C13—C14—C15	0.5 (4)
C1—C2—C3—N1	−175.93 (19)	C13—C14—C15—C16	0.2 (4)
S1—C2—C3—N1	1.9 (2)	C13—C14—C15—C18	−177.7 (2)
C7—N2—C4—N1	3.8 (3)	C14—C15—C16—C17	−0.4 (3)
C7—N2—C4—S1	−174.05 (14)	C18—C15—C16—C17	177.6 (2)
C3—N1—C4—N2	−173.4 (2)	C15—C16—C17—C12	−0.1 (4)
C5—N1—C4—N2	14.7 (3)	C13—C12—C17—C16	0.8 (3)
C3—N1—C4—S1	4.6 (2)	C1—C12—C17—C16	179.59 (19)
C5—N1—C4—S1	−167.20 (14)	C16—C15—C18—O5	2.5 (3)
C2—S1—C4—N2	175.35 (19)	C14—C15—C18—O5	−179.6 (2)
C2—S1—C4—N1	−2.79 (15)	C16—C15—C18—O4	−177.68 (19)
C4—N1—C5—C6	−23.1 (2)	C14—C15—C18—O4	0.2 (3)
C3—N1—C5—C6	165.58 (17)	C24—C19—C20—C21	0.9 (4)
C4—N1—C5—C24	100.8 (2)	C19—C20—C21—C22	0.1 (5)
C3—N1—C5—C24	−70.5 (2)	C20—C21—C22—C23	−0.3 (5)
N1—C5—C6—C7	16.6 (3)	C21—C22—C23—C24	−0.5 (4)
C24—C5—C6—C7	−105.2 (2)	C22—C23—C24—C19	1.5 (4)
N1—C5—C6—C9	−165.65 (17)	C22—C23—C24—C5	−177.9 (2)
C24—C5—C6—C9	72.6 (2)	C20—C19—C24—C23	−1.6 (4)
C9—C6—C7—N2	−178.39 (19)	C20—C19—C24—C5	177.7 (2)
C5—C6—C7—N2	−0.8 (3)	N1—C5—C24—C23	111.4 (2)
C9—C6—C7—C8	3.0 (4)	C6—C5—C24—C23	−127.4 (2)
C5—C6—C7—C8	−179.4 (2)	N1—C5—C24—C19	−67.9 (2)
C4—N2—C7—C6	−10.7 (3)	C6—C5—C24—C19	53.3 (3)
C4—N2—C7—C8	168.12 (19)		

### Hydrogen-bond geometry (Å, °)

**Table:**

*D*—H···*A*	*D*—H	H···*A*	*D*···*A*	*D*—H···*A*
C81—H81···O5^i^	0.93	2.32	3.053 (3)	135
O4—H4···O11^ii^	0.82	1.81	2.618 (3)	170
C17—H17···O3^iii^	0.93	2.43	3.310 (3)	157

Symmetry codes: (i) *x*−1, *y*+1, *z*; (ii) *x*+1, *y*−1, *z*; (iii) −*x*+1, −*y*+1, −*z*.
